# ‘Feeling more like a mechanic’ – A qualitative study on experiences of caries prevention to patients with recurrent cavities among experienced dentists

**DOI:** 10.2340/aos.v83.42271

**Published:** 2024-10-31

**Authors:** Karin Sunnegårdh-Grönberg, Jenny Molin, Håkan Flink, Britt-Marie Lindgren

**Affiliations:** a Dental Public Service, County Council of Västerbotten, Umeå, Sweden; b Department of Odontology, Umeå University, Umeå, Sweden; c Department of Nursing, Umeå University, Umeå, Sweden; d Centre of Clinical Research Västerås, Uppsala University, Västerås, Sweden; e Faculty of Odontology, Malmö University, Malmö, Sweden

**Keywords:** Caries, clinical practice, oral health, dentistry, patient-centered dental care

## Abstract

**Objective:**

To explore experiences of caries prevention in adult patients with recurrent cavities among experienced dentists.

**Method:**

Five focus group discussions consisting of seven men and nine women, 38–61 years of age, and with working experience as dentists between 5 and 35 years, were conducted. The participants represented Public Dental Health Service clinics and private practitioners. Qualitative content analysis was used to analyze data.

**Results:**

The participants emphasized the importance of effective communication and patient engagement in caries prevention. They described their experiences as an endless trail, making fillings. They expressed their inability to take necessary responsibility and being stuck in the dental care system due to various circumstances. The understanding of caries was contradictory, and an inadequate mandate to control time to fulfill their preventive work was evident. They felt responsible to do the best for their patients, but how to share responsibility with colleagues and patients and having enough time for this seemed difficult and unclear. These problems did not motivate to further education in cariology.

**Conclusion:**

The findings underscore the urgent need for improvement in preventive caries treatment and the necessity of allocating sufficient time for dentists to engage in this crucial aspect of their work.

## Introduction

Dental caries, a disease that not only affects an individual’s oral health, general health, and well-being but also incurs significant societal costs, is a global health concern [1–3]. Despite being the most common disease worldwide [4, 5], it is preventable. Recognized as a preventable noncommunicable disease (NCD) [6–8], it shares several behavioral, socioeconomic, and lifestyle factors with other NCDs, such as overweight and diabetes. While there has been a general decline in caries prevalence in several industrialized countries [9–11], the disease remains intimately associated with socioeconomic risk factors, emphasizing the need for preventive measures.

The distinction between caries diagnosis, the recognition of the disease in the individual, and caries detection, which identifies caries lesions on the tooth level, is crucial as it influences the comprehension of caries disease and acts of prevention [12]. Caries lesions, whether in an inactive stage or restored, provide valuable information about episodes of illness and new caries lesions or the progression of a lesion that further destroys the tooth. This underlines the importance of preventive care plans to preserve dental tissue and avoid pulpal exposure, with the goal of retaining the highest number of teeth possible throughout life [7, 13, 14]. Preventing dental caries is beneficial regardless of age, but the impact of preventive efforts, especially in children, can shape future generations’ oral health and healthcare needs [15, 16]. Despite the increased focus on oral health in the Western world, a skewed occurrence of caries disease has emerged [17, 18]. A minority of individuals now account for recurring episodes of caries disease with advanced damages and increasing tooth loss [19, 20]. This skewness poses a challenge in estimating and recognizing actual caries preventive treatment needs and the need for rehabilitation [18]. Inequality in oral health, caused by shortcomings in various caries preventive strategies and an unfair dental system that does not prioritize treatment of illness, has become a stark reality [3]. Paradoxically, more teeth are at risk of developing a cavity than ever before even though improved oral health generally has increased the number of remaining teeth in older age [21]. This underscores the urgent need for improved understanding and treatment strategies to address these disparities, a call to action for all stakeholders in oral health.

In Sweden, caries has declined, and oral health has improved substantially in the past decades. The caries preventive strategies used have been successful in most of the population, but a minority still have troublesome problems with recurrent caries symptoms, that is, cavity formation. In Sweden, there are National Guidelines for Adult Dental Care [22], introduced in 2011 and revised in 2022 [23], which promote effective treatment options and dental care on equal terms. Oral healthcare for adults is provided either by the Swedish Public Dental Health Service or by the private sector. Adult dental care is financed partly by patient charges and partly by the Swedish Social Insurance Agency at several levels. A high-cost threshold is applied, and costs above 3,000 SEK (~ 265 €) subsided by 50% yearly. In Swedish dental public service, caries prevention and nonoperative treatment strategies are targeted to patients at risk, that is, patients with elevated caries risk [23]. These strategies include intensified counseling about oral hygiene and diet, additional fluoride use, and regular dental clinic visits. Nevertheless, a recent study has shown that individuals classified with high caries risk continuously develop caries over a 7-year period that requires a restorative treatment, and only a tiny fraction of these individuals are re-classified as low caries risk [24]. This same study showed that preventive measures and nonoperative treatment were associated with improvements in caries risk assessment and maintenance measures. However, the extent of delivered treatment to high caries-risk individuals was unacceptably low [24]. This is in line with Swedish national data (SKaPa), where the patients receiving restorative treatments due to caries, the proportion with preventive treatment codes registered was below 30% and tended to decrease with older age [11]. Another recent study investigating information and experiences related to caries and its treatment found that caries-active patients reported negative experiences [25, 26]. Preventing caries disease in individuals who develop cavities under circumstances where most people can preserve health has become a challenge for patients and dental health care professionals. Suppose the profession intends to provide successful preventive strategies for caries, leading to equal and improved oral health, even for the most afflicted individuals. In that case, the profession needs a deeper understanding of the patient’s perspectives regarding caries disease and its prevention [27].

## Aim

The aim was to explore experiences of caries prevention in adult patients with recurrent cavities among experienced dentists.

## Materials and methods

This study is based on qualitative data from focus group discussions. Focus group discussion were chosen as the interaction between participants could foster further reflections on personal experiences and thereby enrich data [28]. Data were subjected to qualitative content analysis to highlight the variations, similarities, and differences in the dentists’ experiences [29, 30].

### Participants and data collection

The inclusion criteria were dentists treating adult patients regularly and with a minimum of 5 years’ working experience. Exclusion criteria were if working only within a restricted area of dentistry and not dealing with caries treatment like, for example, implants. Contact was established with four primary clinics in three cities and private practitioners in one of those cities, all located in northern Sweden. The focus groups consisted of dentists from the same primary clinic but active in three cities, except the private practitioners, who constituted their own group. A letter with information about the aim and setting of the study was sent to all dentists before participation. Five focus group discussions were performed with 3–4 individuals participating in each group. The gender distribution among the participants was seven men and nine women, 38–61 years of age, and time of work experience was 5–35 years as working dentists. All participants were educated in Sweden, but not all of them were born and raised in Sweden. A dentist not working at the clinics moderated the focus group discussions. At all times, an observer from the healthcare sector and unfamiliar with dentistry was present.

The opening question in each focus group discussion was ‘Tell us about your experiences of caring for adult patients with recurrent caries?’ to attain free discussion. This was followed by ‘What role does prevention have in treating patients with recurrent cavities and caries activity?’ was raised to attain free discussion. To enrich further discussion, follow-up questions such as: ‘What is caries?’, ‘How is caries treated?’, ‘What affects the outcome of caries treatment?’ ‘Who is responsible for the caries treatment?’, ‘What would strengthen you as a dental therapist?’ and ‘How do dentists experience caries?’ were used when needed. After four focus group discussions were conducted, we noticed that no new information was conveyed; nevertheless we continued with the fifth group as planned. The audio-recorded discussions lasted 45–60 min each and were later transcribed verbatim into text, including notions of non-verbal expressions such as laughter and pauses. A professional translator translated relevant quotations into American English externally.

### Data analysis

The text from the focus group discussions was subjected to qualitative content analysis [28–30]. The method is helpful to structure and process text content and to find underlying themes and meanings. At the beginning of the analysis, the first and last authors listened to the interviews, read the transcripts, and discussed several times to get a sense of the whole. Then, the de-contextualization process started, and each text was divided into meaning units, which are sentences or sections of the texts that relate to the study aim. After condensing the meaning units, that is, shortening the meaning unit without -losing its meaning, they were labeled with a code describing the condensed units meaning. Then, the re-contextualization process began, and the codes were sorted based on their similarities and differences, abstracted, and five categories were formulated. These categories were grouped, abstracted, and interpreted into two sub-themes. A red thread, running through the sub-themes, was interpreted into a theme illustrating dentists’ experiences of caries prevention. During the analysis process, the interpretation of the data was repeatedly discussed among the first, second, and fourth authors, until consensus was reached.

### Ethical considerations

The Regional Ethical Review Board in Umeå, Sweden censored the study, and no further approval was needed (Dnr: 2014/89-31). The participants were informed about the study and that their participation was voluntary. Further, they had the possibility to end their participation at any time without having to provide a reason. All participants gave written consent, and none chose to discontinue participation.

## Results

The analysis of dentist’s experiences of caries prevention in adult patients with recurrent cavities ended up in five categories, two sub-themes, and one main theme (Table 1).

**Table 1:** Overview of categories, sub-themes and main theme revealed in the analysis.

Categories	Sub-themes	Main theme
Shared but still personal responsibility	Being unable to take necessary responsibility	An endless trail
A challenge to engage patients		
Acknowledge the importance of communication		
Inadequate mandate to control time	Being stuck in the dental care system	
Contradictory understanding of caries		

### An endless trail

The participants described their experiences of providing caries prevention as an endless trail. They felt unable to take necessary responsibility and that they were stuck in the dental care system due to various circumstances.

### Being unable to take necessary responsibility

The participants described having a shared but still personal responsibility to provide person-centered caries prevention. Further, they acknowledged the significance of communication as engaging patients in effective self-care was a challenge.

#### Shared but still personal responsibility

The participants discussed their responsibility regarding caries prevention and treatment of patients with recurrent cavities and caries activity. Generally, the participants believed that dental professionals are responsible for the dental care provided. The participants felt that optimized self-care and, in some cases, lifestyle changes targeting caries disease are legitimate ways to counteract recurrent cavities. In addition, the participants also described those dental hygienists provided most of the prevention education, such as by helping patients improve self-care through nonoperative strategies or lifestyle changes. They noted that patients with special needs, such as patients suffering from chronic diseases or the elderly, need special attention. For these populations, most participants believed that dental professionals are obliged to undertake a greater responsibility in treatment.

Although it can be debated what the best treatment plan for recurrent cavities is, most of the participants believed they were responsible for providing a treatment plan, efforts that are often ignored. The participants were frustrated that they were mainly involved in restoring teeth in patients with recurring cavities and less involved in the causative treatment of the disease. They experienced that only restoring teeth in patients with recurring cavities can be a barrier to improved caries prevention in the individual patient. As mentioned, they believed that dental hygienists are the primary health care providers that work with caries prevention for all types of patients irrespective of risk classification or severity of the disease. If a clinic lacks dental hygienists, patients with recurrent cavities will probably not receive even the most basic caries prevention information, as the participants noted that they are occupied with other dental treatments. They described that the distinction between dentists and dental hygienists regarding the responsibility of treatment of patients with recurrent cavities needs to be clarified, even if most of them expressed that dentists are responsible for both treatment plans and the treatment of patients with high caries risk. In one of the focus groups, they expressed:

- ‘No, I mean, basically, I think it’s my responsibility.
- As a dentist, yes
- Yes. That’s how it is.
- That’s how it is.
- But then maybe… it’s easy for the dental hygienists to be the ones who have to, like …
- Do the dirty work’ (FG 2)

They also expressed that even though they are obligated to provide the best possible care for their patients, their patients share responsibility for their well-being and for performing dental prevention measures. The participants discussed the patients’ responsibility to comply with advice. The participants noted that a patient’s inability to control caries disease by following a treatment plan can result in frustration, loss of energy, and even seeing the patient as lacking character. The participants also noted that patients who cannot control their caries disease often blamed their former dentists or their genetics. As expressed in one focus group:

- ‘I don’t think they see themselves as sick. I don’t think they do.
- No.
- They just have a few cavities.
- Yes, exactly. ‘And I’ll go to the dentist to fix this’. (FG 4)

#### A challenge to engage patients

The participants talked about their commitment to helping patients control their caries disease as challenging. They described giving patients the best possible advice regarding optimizing self-care, improving daily routines, or changing behaviors. Many participants found that caries was insufficient to motivate patients to follow through on caries prevention advice. The participants described in detail how they often experienced promoting lifestyle change in diet as more or less impossible to achieve. Instead, they found that changes such as finding a life companion, smoking cessation, losing weight, changing from night-shift to day-shift work, or getting off medications can have a decisive influence on cavity formation and eventually lead to control of caries disease.

The participants discussed their treatment experience in different cases and described a pattern of patient categories and degrees of compliance with given instructions and advice. They described that patients with a disability such as depression or who are fragile in general (e.g. children, the elderly, and refugees) could have different reasons for lack of compliance that often were obvious to understand and accept. Similarly, the participants found that adolescent and adult patients who want to learn more about achieving effective dental self-care are more accessible and motivated to follow through on effective self-care. A majority of the participants experienced caries-active patients who value regular check-ups. These regular check-ups were motivating reminders for the patients to practice self-care intensively. In addition, the participants believed it was their responsibility to help patients understand what restoring caries means. If cavities are restored, the first treatment step is done without feedback about self-care and prevention; patients might believe restorations cure caries disease. The participants noted that this belief was an obstacle to establishing effective self-care in patients with recurrent cavities. Further, they described how this way of working made them feel more like a mechanic than a dentist. One focus group discussed like this:

- ‘Yeah, sure, but now that you’ve emptied your entire register of treatments, what do you do then, but do you give up then, too?
- Yes
- I say that this patient doesn’t cooperate, and then suddenly, it’s the patient’s responsibility to become healthy.
- Yes
- It’s kind of like that
- Yes, that’s the way it is.
- Yes, you do everything you can, and then you don’t have any … You do everything you can, then it’s not exactly like you develop a new strategy immediately maybe
- No, it’s a little hard to think outside the box’ (FG 2)

#### Acknowledge the importance of communication

The participants talked about how they experienced communicating with patients about their caries. Connecting with a patient is seen as a prerequisite to establishing a faithful relationship, and frequent meetings are experienced to improve the relationship with patients. The participants felt they had failed if they could not develop a good relationship with a patient, which often led to further cavities. The participants experienced that how they communicated with a patient significantly influenced whether patients were motivated to follow the advice they received. The participants noted that it took time to understand how to best communicate with their patients. Work experience was also seen as a way to improve communication with patients in certain cases. The participants said they feared they would hurt their patients’ feelings or make them angry when they told them basic facts about their oral hygiene failures. When under stress, the participants noted that acting politely when dealing with patients became more challenging. In addition, if dentists slightly raise their voices due to stress, patients may feel uncomfortable, offended, or vulnerable, disrupting any possible effective communication. The participants experienced nagging and believed that patients did not like this. Some participants felt that motivational interviewing, an evidence-based technique, might improve patient communication. However, they said they were not offered any further education in motivational interviewing because no money was available to enhance communication with patients. One focus group expressed:

‘But…yes, we’ve talked about this, this thing about motivational conversations and all that, and these techniques, that it’s, it’s like, not relevant for us, because it requires education and it requires a long, long time, and we don’t get paid for that either. And not something we currently think patients are prepared to pay for: that we…but what I don’t know, it could be a little better – how about the new dental fee and all that?’ (FG 3)

### Being stuck in the dental care system

The participants described being stuck in the dental system due to various circumstances, meaning they had an inadequate mandate to control their time to fulfill their preventive work. Further, they described a contradictory understanding of caries among dentists, which could be an obstacle to succeeding with caries control in patients with recurrent cavities.

#### Inadequate mandate to control time

The participants discussed terms and conditions associated with dental examinations and treating patients with recurrent cavities. The participants noted that they did not control the time needed to treat patients, perform administrative work, and plan with colleagues. Furthermore, adequately performed examinations and treatments often require more time and cost. They described that the time allocated for examinations only suits fully healthy patients, not patients with problems. According to the participants, insufficient time makes providing sufficient professional contact with patients needing further treatment difficult. The participants emphasized that the lack of time prohibits the transferal of important information about caries diagnostics and causative reasons for the disease.

Further, that lack of time makes it challenging to discuss treatments with colleagues and think about treatment options. Moreover, sometimes, lacking time means doing administrative work during nonworking hours. The participants experienced a lack of time to perform their duties, leading to frustration and feelings of inadequacy. The participants expressed that their mission is to provide their patients with the best treatment possible, which would require more prolonged or more frequent appointments. They also discussed that supporting patients with recurrent cavities requires detailed conversations, reflection, and respect. This approach takes more time, and more time takes more money. In addition, the participants experienced that patients only want to pay for restorations, not information about avoiding cavities. One focus group expressed:

- ‘Although it just feels like this information component that exists now: that is, to charge for
- Yes
- They get furious if you
- Yes, if you charge for it
- Patients don’t like it; they would rather not have the information if they had to pay for it. That’s been my experience, anyway.
- Yeah, yeah, but you sort of have to ease in a little bit, I mean, a little like, you talk while you’re working so that it doesn’t take as much time’. (FG 3)

#### Contradictory understanding of caries

The participants discussed that caries is complex and associated with many risk factors: frequent sugar intake, inefficient tooth brushing, inferior restoration quality, living in exposed socioeconomic areas, low education level, old age, and adolescence. Whether caries disease is hereditary was debated, and the participants’ understanding of this fact was diverse. The participants also discussed whether caries is a ‘true’ disease. If understood as a disease, they believed this would facilitate caries prevention rather than merely relying on symptomatic treatment – that is, restoring teeth. One focus group expressed:

‘This is a disease that can be serious’, etc. That is, you’re really upgrading… don’t just say, ‘You have cavities’, I mean, cavities, in some ways, are purely mechanical. There is a cavity, and I will fill it, yes, of course. We don’t think of the process of a cavity or how it’s formed, but it’s more like fixing the problem. And then they’re filled, and I think, well, it’s okay, and we’ll do this again in 2 years, and then it’s out of this world again and, yeah. So, let’s rank the concept as ‘disease’, meaning cavities are a disease. You can use that word more often’. (FG 4)

Another experience expressed that restoring tooth is an acceptable and reliable caries treatment. The participants experienced that patients do not understand why recurring cavities affect them and that stated questions about caries disease need to be answered correctly and instantly, interactions that require some knowledge that the participants may not possess. This creates insecurity and avoids discussions about detailed mechanisms involved in caries disease. The participants described that their expertise in cariology is limited to what they learned during their undergraduate studies. They experienced that further education in the field is uncommon, even if the area is of interest. They also expressed that other dentistry areas are considered more noble and attractive than cariology and that this may explain why no greater efforts are made to further their education in the field of cariology.

## Discussion

This study explores how experienced dentists deal with caries prevention and treatment in adults with recurrent cavities and caries activity. The findings may describe how a professional attempt to deal with caries disease is obstructed by the dental care system in Sweden. The comprehension of the sub-themes ‘Being unable to take necessary responsibility’ and ‘Being stuck in the dental care system’ give insight into factors that are aggravating successive caries, preventing, and treating one of the most common diseases worldwide [4, 5]. The contradictory understanding of caries as a disease described by the participants in this study is a factor that complicates communication with patients as well as between colleagues. This constitutes a significant barrier to achieving effective caries prevention and treatment. The opinion expressed by several participants that patients with caries disease are not easily motivated to follow through on caries prevention advice reflects this barrier. This difficulty in providing prevention when patients do not want to pay for it is a cause for frustration. The perceived lack of time may result in less accurate examinations, caries diagnostics, and necessary gathering of anamnestic information to pinpoint causative reasons. Discussing treatment plans with colleagues is essential for learning and, ultimately, for treatment success. The participants described feelings of inadequacy when failing in caries treatment. This was related to time pressure or inadequate time allotted to deal with treatments of patients with recurrent cavities and ongoing caries disease. This may imply that the Swedish dental care system cannot deliver effective caries prevention. Dentists in the present study felt ill-equipped regarding caries prevention and a transition to a more preventive focus was seen as difficult. However, the participants did not strive towards further education in the field of cariology. Taken together, it is not surprising that the participants in the actual study felt more like mechanics (doing fillings) than dentists.

Several clinical studies show a low success rate in caries treatment, which underlines the actual findings by means of low efficacy in preventing caries and avoiding recurrent cavities [19, 20, 24, 31–33]. Longitudinal studies indicate that individuals with high caries experience a continuously ongoing progression of caries over time and that the onset of the caries disease starts in childhood [34–36]. The statement in the present study that ‘patients will rather not have the information about prevention if they have to pay for it’ may explain why patients with recurrent cavities fail with effective caries prevention. This finding aligns with others and statements like, for example, ‘Patients don’t pay for the advice!’ expressed by dentists and policymakers [37, 38]. The low interest in prevention shown by patients leads to opinions among professionals that prevention is overemphasized utopic and not always appropriate. Suggesting preventive measures to patients is described as ‘falling on deaf ears’ by others [37, 38]. Caries prevention may have been seen as a waste of time, making the discrepancy between theory and practice visible and may influence newly graduated [37]. Many dentists find restoring cavities the ‘safer’ way to manage caries disease instead of nonoperative treatment and monitoring caries activity [39], which aligns with the present study.

In contrast, there is a clear expert opinion that caries disease should be considered preventable [6–8], but no randomized controlled trials (RCTs) with high-quality evidence confirm this [40]. Simultaneously, most participants in the present study experienced caries, and active patients value regular check-ups. This is in line with others, where patients conclude that caries continue to progress despite exercising preventive measures and also express a strong desire to stop caries progression [25, 26]. These contradictory findings regarding caries disease must be seen as a significant barrier to achieving effective caries prevention. Caries is a major health problem that repeatedly affects approximately 15% of the Swedish population, who do not receive effective caries prevention and treatment [19, 20, 24]. Effective dental care and dental health are ultimately a shared responsibility by society authorities, not least the academy. In the present study, the participants sensed that as experienced dentists, they were responsible for caries treatment and treatment planning for patients with recurrent cavities. At the same time, the statement that dental hygienists ‘Do the dirty work’, which refers to caries prevention, may be surprising. This finding is partly in line with those of Shmarina et al. who explored the dental professionals’ perception of their role in oral health promotion by interviewing dentists and dental hygienists and revealed occupational differences in health promotion versus operative treatment [41]. These findings underline that dental hygienists execute preventive measures to create time so dentists can perform more ‘difficult’ treatments [37, 42]. A common belief is that communicating and motivating patients should be done by dental hygienists alone and not dentists. As also reported by others, preventing and treating caries does not render credit as working with, for example, prosthodontics and orthodontics [38]; another study describes treatment procedures as more attractive than prevention with the statement ‘Prevention not fun or sexy’ [37]. Unfortunately, dental care focuses on surgical and operative procedures on teeth instead of medical intervention [42]. A transition of dental care towards a more holistic and medical approach is needed if the goal is to stop caries progression in individuals with caries disease and recurrent cavities. Today, dental professionals obviously lack the knowledge and skills to carry out effective caries prevention [43]. The inability to communicate leads to mistrust between patients and professionals, and patients feel as though dentists are more interested in making money than providing appropriate and comprehensive dental care to stop caries progression [37, 38, 44]. The participants described their knowledge in cariology as limited, which gave rise to feelings of insecurity and led to avoidance of discussing treatment options with caries patients. Patients with caries and recurrent cavities have empirical knowledge of the disease, which must be recognized by the profession [25, 26]. Patients with recurrent cavities will likely meet dentists with non or minimal interest in caries prevention. The insecurity about caries prevention and treatment described by the participants in the actual study can be linked to the absence of specialized dentists in Sweden’s cariology field. This affects the interest in cariology, the quality of higher education, and the supply of postgraduate courses. However, the most important disadvantage may be the missing input of updated knowledge on caries prevention [45].

### Methodological discussion

The focus group discussions were moderated by an experienced dentist, familiar with the subject studied but not working with the participants. Further, an observer from the healthcare sector, unfamiliar with dentistry was present. On the one hand, shared experiences may facilitate the depth of the phenomenon under study. On the other hand, it may be a risk that colleagues avoid stating the obvious, and therefore it can be seen as positive that the observer had no previous knowledge about dentistry [46]. Our data were rich and revealed a great variation of participants’ lived experiences, both positive and negative, which can indicate that they felt comfortable with the interview situation.

In order to enhance trustworthiness, we have thoroughly described the analysis process. We acknowledge, however, that a text never implies one single meaning, and any interpretation represents just the most probable meaning from a certain perspective [47]. Thus, this is one possible interpretation of dentist’s experiences of caring for people with recurrent caries.

## Conclusion

The findings underscore the urgent need for improvement in preventive caries treatment and the necessity of allocating sufficient time for dentists to engage in this crucial aspect of their work. Negative results and experiences of caries prevention attempts among patients and dental professionals are challenging for the development and interest of the caries disease and its management.

The Swedish dental care system, as it stands, hampers the transition towards effective caries prevention. The lack of development in line with the latest knowledge and scientific evidence is a pressing issue that demands our immediate attention and action. The result of inadequate treatment leads to suffering and vulnerability of the caries sick individuals and is frustrating for the dental team. Preventive caries treatment needs improvement and time to gain interest from dentists.
